# Food protein-induced enterocolitis syndrome: a review of the new guidelines

**DOI:** 10.1186/s40413-017-0182-z

**Published:** 2018-02-07

**Authors:** Stephanie A. Leonard, Valentina Pecora, Alessandro Giovanni Fiocchi, Anna Nowak-Wegrzyn

**Affiliations:** 1 Division of Pediatric Allergy & Immunology, University of California, San Diego, Rady Children’s Hospital San Diego, 3020 Children’s Way, MC 5114, San Diego, CA 92123 USA; 2 Division of Allergy, Pediatric Hospital Bambino Gesù, Piazza Sant’Onofrio 4, 00126 Rome, Italy; 3 Jaffe Food Allergy Institute, Division of Pediatric Allergy, Mount Sinai School of Medicine, New York, NY USA

**Keywords:** Non-IgE mediated food allergy, Food protein-induced enterocolitis syndrome, FPIES, Gastrointestinal food allergy, Cow’s milk allergy

## Abstract

Food protein-induced enterocolitis syndrome (FPIES) is a non IgE-mediated gastrointestinal food allergy that presents with delayed vomiting after ingestion primarily in infants. While the pathophysiology of FPIES is poorly understood, the clinical presentation of acute FPEIS reactions has been well characterized. The first *International Consensus Guidelines for the Diagnosis and Management of Food Protein–induced Enterocolitis Syndrome* were published in 2017 and reviewed epidemiology, clinical presentation, and prognosis of acute and chronic FPIES. The workgroup outlined clinical phenotypes, proposed diagnostic criteria, and made recommendations on management. This article summarizes the guidelines and adds recent updates. FPIES is gaining recognition, however there continues to be delays in diagnosis and misdiagnosis due to overlap of symptoms with over conditions, lack of a diagnostic test, and because some of the common trigger foods are not thought of as allergenic. More research into disease mechanisms and factors influencing differences between populations is needed.

## Background

Food protein-induced enterocolitis syndrome (FPIES) is a non-IgE mediated food allergy characterized by delayed vomiting in infants that was first described in the 1970s. An often underdiagnosed and misdiagnosed condition, FPIES was not associated with its own diagnostic code until 2015. In 2017, the first *International Consensus Guidelines for the Diagnosis and Management of Food Protein–induced Enterocolitis Syndrome* was published by a workgroup of the American Academy of Allergy, Asthma & Immunology [1]. This review of FPIES will serve as a summary of the guidelines, as well as add recent findings since the publication of the guidelines.

Of the two clinical phenotypes, acute FPIES is better defined and easier to recognize than chronic FPIES.

### Acute FPIES

Acute, delayed, repetitive vomiting is the hallmark of acuteFPIES reactions. Patients are often described by parents as looking pale, lethargic, and/or limp. Diarrhea may occur in a subset of patients. The most concerning possible outcome of an acute FPIES reaction is dehydration, which can lead to hypotension and shock if severe.. Compared to IgE-mediated food allergy, anaphylaxis or skin or respiratory symptoms are not seen. Acute FPIES episodes present when the offending food is ingested intermittently or after a period of avoidance. They typically resolve within 24 h and patients are well in between episodes.

### Chronic FPIES

Chronic FPIES is most often seen in infants younger than 4 months on cow’s milk (CM) or soy formula where the offending food is regularly and repeatedly ingested. Symptoms include chronic or intermittent vomiting, diarrhea, and poor weight gain or failure to thrive. Since other gastrointestinal conditions can present similarly, it is the occurrence of acute symptoms after a history of chronic symptoms that confirms the diagnosis of chronic FPIES. Thus, it is common that chronic FPIES is diagnosed either in retrospect or only after ruling out the other possible gastrointestinal disorders.

### Epidemiology

Data on prevalence of FPIES is lacking. One prospective birth cohort study from Israel reported the cumulative incidence of CM FPIES over 2 years to be 0.34% in infants born at a single hospital. [2] A more recent population-wide study in Australia reported the estimated incidence of FPIES to be 15.4/100,000/year in infants less than 2 years old [3]. As awareness of FPIES increases it is probable that future studies will report higher incidence rates.

FPIES usually presents when formula and/or solid foods are introduced, between 2 and 7 months. The most common food triggers are CM, grains, soy (USA, South Korea) and fish (Italy, Spain). Infants on formula typically present earlier than 6 months of age with CM and soy FPIES, compared to infants with solid food FPIES who present at a median age of 5–7 months [1]. Grain FPIES tends to present earlier than fish, egg, and poultry FPIES. Depending on the population, between 65 and 80% of patients have FPIES to a single food, most often CM, while 5–10% have reacted to more than 3 foods [1]. In a large U.S. case series, 5% of patients reacted to as many as 6 foods [4]. In the Australian birth cohort, infants with multiple versus single food group FPIES were younger at first presentation (mean 4.6 vs. 5.8 months, *P* = .001) and were more likely to have FPIES to fruits, vegetables, or both (66% vs. 21%, *P* < .0001) [3]. In addition, infants exclusively breast-fed for more than 4 months appeared to have lower rates of multiple food group FPIES (23% vs. 36%, *P* = .06).

Atopic conditions are commonly seen in FPIES patients in some populations. Between 11 and 57% of patients had eczema in U.S. and Australian populations, while only 0–9% did in Korean, Israeli, and Italian populations [1]. Also in U.S. and Australian populations, food sensitization to foods other than FPIES triggers was seen in 16–39% of FPIES patients.

In the large Israeli birth cohort, CM FPIES was associated with birth by Caesarean section and Jewish religion [2]. It was not associated with gestational age, maternal age, number of siblings, maternal dairy consumption, or age of introduction of CM. Other studies have not reported on perinatal risk factors.

### Clinical presentation

In acute FPIES reactions, repetitive vomiting (>1 and up to >10 times has been reported by families) typically develops 1–4 h after ingestion of the food allergen and is often accompanied by lethargy, pallor, and/or limpness. Diarrhea may occur within 5–10 h in a subset of patients. Symptoms can lead to dehydration and if severe, hypotension and hypovolemic shock requiring emergency care. Hypothermia, methemoglobinemia, and acidemia have also been reported, and patients may appear to have sepsis.

Chronic FPIES typically presents with chronic or intermittent vomiting, diarrhea, and poor weight gain or failure to thrive. These symptoms can sometimes lead to dehydration and shock, and hypoalbuminemia may be seen. Chronic FPIES symptoms resolve with elimination, however when the food trigger is re-ingested at a later time, a picture of acute FPIES occurs.

Clinical phenotype is affected by age of onset, nationality, frequency of allergen ingestion, and presence of IgE-mediated food allergy. Infants less than 2 months of age diagnosed with CM or soy FPIES are more likely to present with diarrhea, blood in stool and failure to thrive in addition to vomiting as compared to those presenting later [1]. Older infants are more likely to present with vomiting only and no diarrhea. Symptoms consistent with FPIES may present in older children and adults with delayed vomiting after ingestion of fish, shellfish or egg.

Combined CM/soy FPIES is common in U.S. populations (20–40%), but not in Australia, Italy, or Israel. [1] The likelihood of having combined CM/soy FPIES appears higher if symptoms begin within the first month of life. Rice is the most common solid food FPIES, except in Italy and Spain. Combined rice/oat FPIES is common (up to 1/3) in U.S. and Australian populations. Other food triggers commonly seen together are detailed in Table 1 [1]. Fish FPIES is common in Italy and Spain, but not other countries. Egg is the third most common trigger in Australia but is reported less frequently from the other countries [3].

**Table 1:** Common food co-allergies in children with FPIES [1]

FPIES to	Clinical cross-reactivity/co-allergy	Observed Occurrence^a^
Cow’s milk	Soy	<30–40%
	Any solid food	<16%
Soy	Cow’s Milk	<30–40%
	Any solid food	<16%
Solid food (any)	Another solid food	<44%
	Cow’s milk or soy	<25%
Legumes^a^	Soy	<80%
Grains: rice, oats, etc.^a^	Other grains (including rice)	about 50%
Poultry^a^	Other poultry	<40%

^a^where a child already tolerates a food type in a particular group (e.g. beans), clinical reactions to other members of the **same** group (e.g. other legumes) are unlikely. Caution is warranted in interpreting these data as they were derived from single centers and from patient populations skewed towards the more severe phenotype of FPIES and may overestimate the actual risk of co-allergy

A Japanese cohort reported FPIES symptoms after breastfeeding, presumably because of the food protein from maternal ingestion in the breastmilk, in 10% of patients and the Australia cohort reported the same in 5% [3, 5]. Only case reports have been noted in other populations. Chronic FPIES has been diagnosed more often in Japan and Korea than reported in other countries. Differences in population phenotypes may be related to the degree of associated atopy, breastfeeding and dietary practices, and genetics.

### Pathophysiology

The pathophysiology of FPIES is not well understand but it is thought that a reaction against food protein leads to gut inflammation, which causes increased intestinal permeability and a fluid shift resulting in vomiting, diarrhea, and shock. Non-specific inflammation has been found in the colon and ileum by endoscopy and biopsy. Antigen-specific T cells and inflammatory cytokines have been reported. A recent study confirmed the lack of serum humoral response in FPIES but noted increased serum IL-8 and tryptase in active FPIES, which could suggest neutrophil and mast cell involvement [6]. A subsequent study by the same group demonstrated activation of innate immune cells from whole blood after positive FPIES OFCs, including monocytes, neutrophils, natural killer cells, and eosinophils [7]. Additional studies are needed to elucidate mechanisms and food specificity of these observations.

### Diagnosis

A diagnostic laboratory test is not available for FPIES at this time, reflecting obscure pathophysiology. Instead, diagnosis is based on a clinical history consistent with typical signs and symptoms, and resolution of symptoms with avoidance of the suspected food trigger. It is common for there to be a delay in diagnosis due to the nonspecific symptoms, the lack of familiarity with FPIES, and because some of the solid food triggers, such as rice and oat, are not typically considered allergenic. Most often acute FPIES is misdiagnosed as acute viral gastroenteritis or sepsis, and often patients presenting to the emergency department or hospitalized end up getting an extensive work-up. FPIES symptoms should be reproducible if there is re-exposure to the food trigger. Oral food challenges (OFCs) may be needed if the diagnosis is not clear. It is important to consider a broad differential diagnosis for acute vomiting, including infectious gastroenteritis, sepsis, necrotizing enterocolitis, anaphylaxis, metabolic disorders, severe lactose intolerance, neurologic disorders (e.g. cyclic vomiting), gastroesophageal reflux disease, and gastrointestinal obstruction (Table 2) [1].

**Table 2:** Differential diagnosis of FPIES [1]

Condition	Features that may distinguish from FPIES
Infectious gastroenteritis (e.g. viral, bacterial)	Single episode of illness, fever, sick contacts
Sepsis	Fluid resuscitation alone not effective
Necrotizing enterocolitis (NEC)	Newborns and younger infants, rapid escalation of symptoms, bloody stools, shock, intramural gas on abdominal radiographs
Anaphylaxis	Symptoms begin within minutes to 2 h of exposure, positive IgE testing, usually other manifestations (e.g. urticaria)
Food aversion	Look at the familial context
Inborn errors of metabolism: Urea cycle defects, Hereditary fructose intolerance, hyperammoiniemic syndromes, propionic /methylmalonic aciduria, beta-oxydations defects, hyperinsulinism-hyperammonemia syndrome, Pyruvate dehydrogenase deficiency, mitochondrial disorders, maple syrup urine disease, ketothiolase deficiency.	Developmental delay, neurologic manifestations, organomegaly, reaction to fruits
Lactose intolerance	In severe form, gas, bloating, cramps, diarrhea, borborygmi and vomiting following ingestion of liquid milk and large doses of dairy products with lactose
Neurologic disorders (e.g. cyclic vomiting)	No relation to specific food intake
Gastrointestinal reflux disease	Emesis more chronic and not usually severe (i.e. does not lead to dehydration), only upper GI symptoms present
Hirschsprung’s disease	Delay in passage of the first meconium, marked abdominal distention
Food protein-induced enteropathy	Symptoms usually not temporarily associated with specific food intake, symptoms more chronic than episodic, vomiting less severe, most commonly implicated foods cow milk, soy, wheat, egg white
Eosinophilic gastroenteropathies (e.g. eosinophilic esophagitis, eosinophilic gastroenteritis)	Usually not associated with specific food intake, symptoms more chronic than episodic, vomiting less severe, more likely to have positive IgE tests
Celiac disease	No temporal relationship between symptoms and specific food intake; progressive malabsorption; celiac serology is positive
Immune enteropathies (e.g. inflammatory bowel disease, autoimmune enteropathy, immunodeficiency)	Rare in infancy, not related to specific food intake
Obstructive problems (e.g. malrotation, Ladd’s bands, volvulus)	Not related to specific food intake, evidence of obstruction on radiological studies
Coagulation defects	No relation to specific food intake
Alpha1-antitrypsine deficiency	No relation to specific food intake; hepatic involvement
Primary immunodeficiencies	No relation to specific food intake; intestinal symptoms, frequent infections.

History is often adequate to diagnose acute FPIES and identify the food trigger. Timing of symptoms after ingestion is important. For example, immediate vomiting in an infant after ingestion of a new food may be indicative of IgE-mediated food allergy while repetitive vomiting more than 2 h after ingestion of an intermittently ingested food is more indicative of FPIES. If the history is clear with repeated episodes of delayed vomiting to the same or more than one identified food, the risks of OFCs may outweigh the benefits, and a presumed clinical diagnosis can be made with OFC.

History alone may not be enough to diagnose chronic FPIES. Supervised OFCs of suspected food triggers after elimination might be warranted. Symptoms can normalize as soon as 2 days after removal of the trigger food. Evaluation for other conditions with possible use of endoscopy and biopsy is important, especially if symptoms are severe and/or failure to thrive is present. Overall, however, radiographic testing or endoscopy is not recommended as routine evaluation if FPIES is suspected, since findings in FPIES have been nonspecific [1]. The differential diagnosis for chronic FPIES may include eosinophilic gastroenteropathies, celiac disease, and inflammatory bowel disorders (Table 2) [1].

While no laboratory testing is specific to FPIES, laboratory findings can help support a diagnosis or rule out other conditions. In acute FPIES, an increased blood neutrophil count >1500 cells/mL above baseline may be seen that peaks about 6 h after food ingestion. In one case series thrombocytosis was present in 65% of reactions [8]. If severe, methemoglobuinemia and metabolic acidosis may be present in both acute and chronic FPIES. Recently, some reports emphasized the increase of CRP after the oral food challenge or during an acute FPIES reaction and in particular, a correlation between CRP levels and the degree of reaction severity was observed. [9–11] Increased levels of blood lactate dehydrogenase (LDH) and serum glutamic oxaloacetic transaminase (SGOT) in resting conditions were observed in FPIES patients, suggesting the presence of some preexisting intestinal cellular damage. Diarrhea or blood, leukocytes, or increased carbohydrates in stool may be detected during reactions. In one study, gastric aspirates collected pre and post challenge showed an increase of 10 leukocytes/high-powered field about 3 h after food ingestion of a positive challenge, which was not seen in subjects who did not react [12]. Some of these tests may not be practical in a clinical setting but can be observed during supervised OFCs or in a research setting.

In chronic FPIES, anemia from chronic blood loss in the stool and hypoalbuminemia may be seen. Peripheral neutrophilia and eosinophilia have also been noted. Diarrhea or blood, neutrophils, eosinophils, Charcot Leyden crystals, or reducing substances in stool may be detected. Stool cultures should be negative for organisms and parasites. It is not recommended that stool tests be used to diagnose FPIES since the findings are nonspecific [1].

Most FPIES patients have negative skin prick testing and undetectable specific IgE levels to their trigger food. Coexisting IgE sensitization to FPIES triggers have been reported in up to 8–25% of patients in the U.S. and Australia [1]. In one Japanese cohort of CM FPIES 47% had detectable milk-specific IgE levels [9]. Since it appears that those with IgE sensitization to their FPIES trigger food may have a more protracted FPIES course, testing may be considered based on clinical history or history of atopy (eczema or other IgE-mediated food allergy to other foods). While not recommended for initial diagnosis, it could be important to perform testing for decision-making, for example whether to delay an OFC if testing is significantly positive, or whether to perform an OFC gradually with medications and supplies to treat an IgE-mediated reaction as well. In a large U.S. cohort, 41% of patients with CM FPIES transformed into an IgE-mediated phenotype over time, and those with CM sensitization were more likely to have their FPIES persist beyond 3 years of age [13]. Sensitization to other foods did not appear to affect the development of tolerance. A shift from IgE-mediated food allergy to FPIES has also been reported. [11, 14] Atopy patch testing for FPIES trigger foods has not been supported by data and is not recommended in the evaluation of FPIES.

### Diagnostic criteria for acute FPIES

The guidelines propose criteria that if the major criterion and at least 3 minor are met then it is a likely FPIES diagnosis (Table 3) [1]. The major criterion is vomiting 1–4 h after ingestion of a food with absence of IgE-mediated skin or respiratory symptoms. The minor criteria include: a second or more episode of repetitive vomiting after ingestion of the same suspect food, repetitive vomiting episodes 1–4 h after eating a different food, extreme lethargy, marked pallor, need for an emergency department visit, need for intravenous fluids, diarrhea within 24 h (usually 5–10 h after food ingestion), hypotension, and hypothermia. It is recommended that if only a single episode occurs to consider an OFC since acute gastroenteritis is common in infants.

**Table 3:** Diagnostic criteria for patients presenting with possible FPIES [1]

**Acute FPIES**	
**Major criterion:** Vomiting in the 1–4 h period after ingestion of the suspect food and the absence of classic IgE-mediated allergic skin or respiratory symptoms	**Minor criteria:** 1. A second (or more) episode of repetitive vomiting after eating the same suspect food 2. Repetitive vomiting episode 1–4 h after eating a different food 3. Extreme lethargy with any suspected reaction 4. Marked pallor with any suspected reaction 5. Need for emergency room visit with any suspected reaction 6. Need for intravenous fluid support with any suspected reaction 7. Diarrhea in 24 h (usually 5–10 h) 8. Hypotension 9. Hypothermia
**The diagnosis of FPIES requires that a patient meets the major criterion and at least 3 minor criteria.** If only a single episode has occurred, a diagnostic oral food challenge should be strongly considered to confirm the diagnosis, especially since viral gastroenteritis is so common in this age group. Further, while not a criteria for diagnosis, it is important to recognize that acute FPIES reactions will typically completely resolve over a matter of hours, compared to the usual several day time course of gastroenteritis. The patient should be asymptomatic and growing normally when the offending food is eliminated from the diet.	
**Chronic FPIES**	
**Severe presentation**: when the offending food is ingested in on a regular basis [e.g., infant formula]. Intermittent but progressive vomiting and diarrhea (occasionally with blood) develop, sometimes with dehydration and metabolic acidosis. **Milder presentation**: lower doses of the problem food (e.g. solid foods or food allergens in breast milk) lead to intermittent vomiting, and/or diarrhea, usually with poor weight gain/ failure to thrive, but without dehydration or metabolic acidosis.	The most important criterion for chronic FPIES diagnosis is resolution of the symptoms within days following elimination of the offending food(s) and acute recurrence of symptoms when the food is reintroduced, onset of vomiting in 1–4 h, diarrhea in 24 h (usually 5–10 h). Without confirmatory challenge, the diagnosis of chronic FPIES remains presumptive.

### Diagnostic criteria for chronic FPIES

The guidelines do not have suggested criteria for chronic FPIES (Table 3) [1]. The diagnosis should be considered if there is intermittent but progressive vomiting and diarrhea (with or without blood) in an infant, particularly in infants on regular CM or soy formula. There is often poor weight gain and possibly failure to thrive, particularly in very young infants. If severe, symptoms can lead to dehydration and metabolic acidosis. Symptoms typically resolve within days of avoidance and reintroduction results in an acute FPIES reaction. Without an OFC or an acute reaction, the diagnosis of FPIES is presumptive.

### Oral food challenges

OFCs are the gold standard in diagnosis of FPIES if the food trigger cannot be identified by history alone, if the timing of symptoms is atypical for a presumed FPIES reaction (for example, repetitive vomiting immediately after ingestion with negative testing), or if symptoms persist after avoidance of the suspected food trigger. OFCs are also useful to assess whether FPIES has been outgrown. Criteria for determining a positive FPIES challenge are presented in Table 4 [1]. There is no standard protocol for an FPIES OFC, as this procedure has not been studied. Placement of peripheral intravenous access is suggested particularly in patients with a history of severe reactions, emergency department treatment, or hospitalization. It has been reported that 15% of FPIES reactions present with hypotension and hypovolemic shock and 45–95% of FPIES OFC reactions have been treated with intravenous fluids, steroids or both [1]. Some OFC reactions may resolve with oral rehydration, but it is advised to have the means to administer intravenous fluids readily available. Due to the potential for a severe reaction home reintroduction of the suspected trigger food is not recommended for suspected FPIES triggers. If FPIES reactions occur at home, either after accidental ingestion or to a new food, oral rehydration may be attempted if vomiting has been minimal (1–2 times) and there is little to no lethargy (Table 5) [1]. If vomiting is repetitive (> 3 times) and there is moderate to severe lethargy, recommendations are to access emergency medical services for intravenous hydration and other support.

**Table 4:** Diagnostic criteria for the interpretation of oral food challenges in patients with a history of possible or confirmed FPIES [1]

Major criterion	Minor criteria
Vomiting in the 1–4 h period after ingestion of the suspect food and the absence of classic IgE-mediated allergic skin or respiratory symptoms	1. Lethargy 2. Pallor 3. Diarrhea in 5–10 h after food ingestion 4. Hypotension 5. Hypothermia 6. Increased neutrophil count of at least 1500 neutrophils above the baseline count
**The OFC will be considered diagnostic of FPIES, i.e. positive, if the major criterion is met with at least two minor criteria.** However, we would suggest two important caveats to these criteria: 1) with the rapid use of ondansetron, many of the minor criteria, such as repetitive vomiting, pallor and lethargy may be averted; and 2) not all facilities performing challenges have the ability to perform neutrophil counts in a timely manner. Therefore, the treating physician may decide that a challenge be considered diagnostic in some instances even if only the major criterion was met. However, in challenges performed for research purposes, stringent criteria for challenge positivity should be adhered to.

**Table 5:** Management of acute FPIES episode at home [1]

Current episode	Mild ^a,b^	Moderate-severe
Symptoms	1–2 episodes of emesis No or mild lethargy	More than 3 episodes of emesis and moderate-severe lethargy
Management	Attempt oral re-hydration at home (e.g., breast-feeding or clear fluids)	Call 911 or go to the emergency room

^a^Child **with history of severe FPIES reaction**: call 911 or go to the emergency department if the triggering food was definitely ingested, even in the absence of symptoms or with any symptoms regardless of severity

^b^Child **with no history of severe FPIES reaction**

Some FPIES OFC protocols administer one full dose and monitor for 4–6 h:most often a dose 0.06–0.6 g/kg of food protein (maximum 4 g of protein; 10 g of total food or 100 ml of liquid) is given in 2–3 equal doses over 30–60 min [1]. The dose, time between doses, and monitoring period can be individualized based on a patient’s history. If an initial low doses is used, for example due to a severe reaction in the past, and no symptoms develop after a few hours, then it is recommended to follow this with ingestion of an age appropriate serving and further monitoring for at least 4 h.

The guidelines include determination of OFC positivity if the major criterion and 2 or more minor criteria are met [1]. If OFCs are performed in a controlled environment and symptoms are treated immediately, just the major criterion may be considered diagnostic. The major criterion is vomiting 1–4 h after ingestion of the suspect food without IgE-mediated allergic skin or respiratory symptoms. The minor criteria include: lethargy, pallor, diarrhea (5–10 h after ingestion), hypotension, hypothermia, and neutrophilia >1500 cells/mL above baseline. Obtaining a baseline and post challenge complete blood count (CBC) may be more useful in research since this test would not be diagnostic on its own.

### Management of Reactions

Acute FPIES reactions should be managed individually according to severity. The guidelines provide recommendations for treatment reported in Table 6 [1]. Mild reactions can resolve with oral rehydration. Moderate to severe reactions require aggressive fluid resuscitation (10–20 ml/kg normal saline boluses) with repeated boluses and maintenance fluids with dextrose as needed. Although there is no data to support the use of steroids in FPIES reactions, a single dose of IV methylprednisolone (1 mg/kg, max 60-80 mg) may be given for presumed inflammation in severe reactions. Intravenous vasopressors may be required for treatment of shock in very severe reactions. Oxygen, respiratory support, and corrections for academia and methemoglobulinemia may be used as needed. Epinephrine is not recommended as routine treatment of FPIES reactions because it has no effect on emesis although epinephrine autoinjectors should be prescribed for those with IgE sensitization or IgE-mediated food allergy if they are at risk for anaphylaxis.

**Table 6:** Management of acute FPIES episode at the medical facility [1]

Presenting Symptoms		
Mild	Moderate	Severe
Symptoms		
1–2 episodes of emesis No lethargy	> 3 episodes of emesis and mild lethargy	>3 episodes of emesis, with severe lethargy, hypotonia, ashen or cyanotic appearance
Management		
1. Attempt oral re-hydration (e.g., breast-feeding or clear fluids) 2. If age 6 months and older: Consider ondansetron intramuscular 0.15 mg/kg/dose, maximum 16 mg/dose 3. Monitor for resolution about 4–6 h from the onset of a reaction	1. If age older than 6 months: administer ondansetron intramuscular 0.15 mg/kg/dose, maximum 16 mg/dose 2. Consider placing a peripheral intravenous line for normal saline bolus 20 ml/kg, repeat as needed 3. Transfer the patient to the emergency department or intensive care unit in case of persistent or severe hypotension, shock, extreme lethargy, or respiratory distress 4.Monitor vital signs 5. Monitor for resolution at least 4–6 h from the onset of a reaction 6. Discharge home if patient is able to tolerate clear liquids	1. Place a peripheral intravenous line and administer normal saline bolus 20 ml/kg rapidly, repeat as needed to correct hypotension 2. If age 6 months and older: administer intravenous ondansetron 0.15 mg/kg/dose, maximum 16 mg/dose 3. If placement of intravenous line is delayed due to difficult access and age is 6 months or older administer ondansetron intramuscular 0.15 mg/kg/dose, maximum 16 mg/dose 4. Consider administering intravenous methylprednisolone 1 mg/kg, maximum 60 to 80 mg/dose 5. Monitor and correct acid base and electrolyte abnormalities 6. Correct methemoglobinemia if present 7. Monitor vital signs 8. Discharge after 4–6 h from the onset of a reaction when the patient is back to baseline and is tolerating oral fluids 9. Transfer the patient to the emergency department or intensive care unit for further management in case of persistent or severe hypotension, shock, extreme lethargy, respiratory distress

Strong consideration should be lent in performing food challenges in children with history of severe FPIES in the hospital or other monitored setting with immediate availability of intravenous resuscitation.

Oral challenges in the physician’s office can be considered in patients with no history of a severe FPIES reaction, although caution should be urged as there are no data that can predict future severity of FPIES reactions

Two cases series reported that treatment with intravenous or intramuscular ondansetron was associated with cessation of vomiting during FPIES OFC reactions and thus can be considered an adjunctive therapy [15, 16]. Ondansetron is a serotonin 5-HT_3_ receptor antagonist and its successful use in FPIES suggests a possible neuroimmune mechanism. Placebo controlled trials are needed to assess the efficacy and safety of ondansetron, and caution should be used in patients with heart disease due to its potential to prolong the QT interval.

### Dietary management

Long-term management of FPIES includes avoidance of the trigger food(s), dietary and nutritional monitoring, treatment of reactions in case of accidental ingestion or new trigger foods, and assessing for resolution. For infants with CM or soy FPIES, breastfeeding or use of an extensively hydrolyzed casein formula is encouraged. Since combined CM/soy FPIES is not common in all populations supervised introduction of one or the other can be considered. Most infants will tolerate a hypoallergenic formula; however 10–20% may require an elemental formula [1]. While there are case reports of baked milk and egg tolerance in milk and egg FPIES, tolerance of baked or processed CM is not well understood and avoidance is recommended unless the patient is already tolerating these forms or they are introduced under physician supervision. Due to cross-reactivity of similar proteins, patients with CM FPIES should also avoid goat’s and sheep’s milk.

Maternal avoidance of an infant’s FPIES triggers while breastfeeding is not recommended if the infant is thriving and asymptomatic [1]. Mothers should avoid trigger food(s) if a reaction occurs after breastfeeding or if there is failure to thrive. If symptoms do not resolve, discontinuing breastfeeding and introducing a hypoallergenic formula should be considered.

Infants with milk or soy FPIES are more likely to have FPIES to solid food, most commonly rice or oat [1]. Therefore dietary guidance for young infants may include starting with fruits and vegetables around age 6 months, and then red meat and cereals. Diets can typically be expanded if infants are tolerating a variety of complementary foods. The guidelines outline low-, moderate-, and high-risk foods to consider when expanding diets in infants with FPIES [1]. (Table 7) Families of infants with CM or soy FPIES may be hesitant to introduce solid foods initially or additional foods if they have experienced severe reactions. Supervised introduction should be considered to avoid unnecessary avoidance and encourage variety. This can be done as a mixture of several solids followed by gradual build up to appropriate servings at home. Alternatively, OFCs to key foods can be performed with the observation that if infants tolerate one food from a food group they are likely to tolerate others in the same group.

**Table 7:** Empiric guidelines for selecting weaning foods in infants with FPIES [1]

Ages and Stages	Lower risk foods^c^	Moderate risk foods^c^	Higher risk foods^c^
**4–6 months (as per AAP, CoN)** If developmentally appropriate and safe and nutritious foods are available. ➢ Begin with smooth, thin, purees and progress to thicker purees ➢ Choose foods that are high in iron ➢ Add vegetables and fruits	**Vegetables**		
	Broccoli, cauliflower, parsnip, turnip, pumpkin	Squash, carrot, white potato, green bean (legume)	Sweet potato, green pea (legume)
**6 months (as per WHO)** Complementary feeding should begin no later than 6 months of age. ➢ In the breast fed infant, high iron foods or supplemental iron (1 mg/kg/day) is suggested by 6 months of age. ➢ Continue to expand variety of fruits, vegetables, legumes, grains, meats and other foods as tolerated.	**Fruits**		
	Blueberries, strawberries, plum, watermelon, peach, avocado	Apple, pear, orange	Banana
**8 months** of age or when developmentally appropriate. ➢ Offer soft-cooked and bite-and-dissolve textures from around 8 months of age or as tolerated by infant.	**High iron foods**		
	Lamb, fortified quinoa cereal, millet	Beef, fortified grits and corn cereal, wheat (whole wheat and fortified), fortified barley cereal	Higher iron foods: Fortified, infant rice and oat cereals.
**12 months of age** or when developmentally appropriate. ➢ Offer modified tolerated foods from the family table-chopped meats, soft cooked vegetables, grains and fruits.	**Other**		
	Tree nuts and seed butters^c^ (sesame, sunflower, etc.) ^c^Thinned with water or infant puree for appropriate infant texture and to prevent choking	Peanut, other legumes (other than green pea)	Milk, soy, poultry, egg, fish

This table should be considered in the context of the following notes:

^a^Exclusive breast feeding until 4–6 months of age and continuing breast feeding through the first year of life or longer as long as mutually desired by both mother and child [17]

^b^If an infant tolerates a variety of early foods, subsequent introduction may be more liberal. Additionally, tolerance to one food in a food group (green pea) is considered as a favorable prognostic indicator for tolerance of other foods from the same group (legumes) [18]

*AAP*, *CoN* American Academy of Pediatrics, Committee on Nutrition, *WHO* World Health Organization

^c^Risk assessment is based on the clinical experience and the published reports of FPIES triggers

Infants with FPIES are at risk for nutritional and developmental deficiencies due to dietary restrictions and delay in introductions. Nutritional consultation is highly recommended to assist with avoidance and advancement of the diet [1]. Generally it is recommended that one food be introduced at a time with 4 days in between adding a new food to be able to monitor for symptoms. Foods that enhance developmental skills, such as those with different texture potentials (pureed, soft cooked, baked), are recommended to help prevent food aversions and delays in food acceptance and feeding skills. Growth should be normal in FPIES patients who are avoiding their trigger food and are asymptomatic. Multiple food FPIES or feeding difficulties might put some infants at risk for poor growth and they should be monitored closely.

### Natural history

The development of tolerance varies based on food and nationality. Tolerance to milk and soy is achieved earlier than to grain or other solid foods. The average age for grain tolerance is 35 months; in U.S. populations the median tolerance age for rice is 4.7 years and for oat 4.0 years [1]. The average age for soy tolerance is 12 months (range 6 months to >22 years). Age at tolerance to CM varies as widely, with a significant number by age 12 months in Korea, >90% by 3 years in Israel, a median of 6.7 years in the U.S., and 75% in the United Kingdom.

It is recommended that FPIES patients be evaluated regularly based on age and food to see if they are still allergic. Tolerance has not been systematically studied and how often patients are reassessed depends on country, food importance, and individual preference. In the U.S. it is practice to recommend waiting 12–18 months since last reaction to consider a food challenge [1]. It is unknown whether older children and adults outgrow seafood FPIES but monitoring should be considered. Reintroduction of the FPIES food should be under physician supervision as a formal OFC or supervised feeding. Reintroduction at home is not encouraged but may depend on access to emergency care, caregiver comfort, and severity of past reactions.

## Conclusions

Awareness is increasing for FPIES, a non-IgE mediated food allergy characterized by delayed vomiting that typically presents in infancy. The most common casual foods are milk, soy and grains (rice, oat). FPIES phenotype depends on age of onset, trigger foods, and nationality. Diagnosis of FPIES is clinical and OFCs may be used if the diagnosis is unclear. Criteria for diagnosing acute FPIES, which occur when the food is ingested intermittently, are proposed in the First International Consensus Guidelines. Currently there are no criteria for diagnosing chronic FPIES, which typically presents with intermittent but progressive vomiting and diarrhea in young infants with regular ingestion of milk or soy formula. It is important to rule out other gastrointestinal disorder when considering chronic FPIES. Treatment of FPIES is supportive and focuses on removal of offending food and management of vomiting, dehydration and shock. Variety and dietary advancement is vital to an infant’s nutrition and development and can be challenging in infants with multiple food FPIES or feeding issues. FPIES patients should be monitored regularly for the development of tolerance and foods should be introduced using physician-supervised challenges. Additional studies are needed to increase understanding of pathophysiology as well as mechanisms of different phenotypes.

## Abbreviations

CBC: Complete blood count
CM: Cow’s milk
FPIES: Food protein-induced enterocolitis syndrome
LDH: Lactate dehydrogenase
OFC: Oral food challenge
SGOT: Serum glutamic oxaloacetic transaminase
